# Epigenetic Segregation of Microbial Genomes from Complex Samples Using Restriction Endonucleases HpaII and McrB

**DOI:** 10.1371/journal.pone.0146064

**Published:** 2016-01-04

**Authors:** Guohong Liu, Christopher Q. Weston, Long K. Pham, Shannon Waltz, Helen Barnes, Paula King, Dan Sphar, Robert T. Yamamoto, R. Allyn Forsyth

**Affiliations:** 1 FLIR Systems, Inc., La Jolla, California, 92037, United States of America; 2 Singlera Genomics, Inc., La Jolla, California, 92037, United States of America; 3 Zova Systems, LLC, San Diego, California, 92129, United States of America; 4 San Diego State University, San Diego, California, United States of America; The Scripps Research Institute and Sorrento Therapeutics, Inc., UNITED STATES

## Abstract

We describe continuing work to develop restriction endonucleases as tools to enrich targeted genomes of interest from diverse populations. Two approaches were developed in parallel to segregate genomic DNA based on cytosine methylation. First, the methyl-sensitive endonuclease HpaII was used to bind non-CG methylated DNA. Second, a truncated fragment of McrB was used to bind CpG methylated DNA. Enrichment levels of microbial genomes can exceed 100-fold with HpaII allowing improved genomic detection and coverage of otherwise trace microbial genomes from sputum. Additionally, we observe interesting enrichment results that correlate with the methylation states not only of bacteria, but of fungi, viruses, a protist and plants. The methods presented here offer promise for testing biological samples for pathogens and global analysis of population methylomes.

## Introduction

Next Generation Sequencing (NGS) has expanded our perception of microbial diversity particularly in the human microbiome [1] which plays roles in diverse clinical conditions such as obesity, allergies and cancer [2–5]. Polymicrobial infections [6] and the causative agent of more than twenty disease outbreaks have been identified using NGS in the last few years [7]. A key advantage of NGS in these studies is the non-hypothesis driven approach which allows detection of novel pathogens where primers or probes would have missed the causative agent [8, 9], as well as characterization of unexpected genes such as virulence factors in *Staphylococcus aureus* [10] and macrolide resistance in *Mycobacterium tuberculosis* [11].

Nevertheless for most clinical sample DNA preparations, microbes, particularly pathogens, are typically present at trace levels resulting in inefficiently sequencing a vast majority of host DNA rather than the desired microbiome or causative pathogen. Techniques to improve targeted sequencing have been developed but recent epigenetic methods to segregate target genomes [12–14] have the advantage of enriching nearly whole genomes for sequencing. However, the epigenome of only a small number of bacterial species has been well defined [15–17], and epigenomes of protists, fungi and viruses remain poorly characterized.

We report the development of two complementary methods to enrich broad classes of microbial genomes including DNA viruses and fungi from human backgrounds. First, the restriction endonuclease HpaII was used under conditions where it does not digest DNA but will bind to its non-methylated target CCGG pattern which is widely present in the bacterial kingdom. Binding and enrichment capability was loosely related to the GC content of the microbe but HpaII showed little binding in the human genome where CCGG motifs are typically methylated which is entirely consistent with HpaII digestion activity. HpaII mediated enrichment, applied to *in vitro* genomic mixtures as well as DNA isolated from sputum showed greater than 100-fold enrichment of many microbial genomes. For the second method, the N-terminal DNA-binding domain of the Type IV methyl directed restriction endonuclease McrB (McrB-N) was used to bind and segregate human DNA from *in vitro* genomic mixtures. McrB-N has a low affinity for non-CpG methylated DNA but high affinity for the recognition motif RmC(N)_40-2000_RmC [18] which appears to involve binding of several McrB molecules [19]. McrB-N depleted genomic mixtures resulting in a broad 8-fold enrichment of microbial genomes. Our results support the ability to enrich microbial genomes from complex samples such as sputum and to help categorize the methylation state of poorly studied genomes.

## Materials and Methods

Genomic DNA was obtained from the ATCC with the following exceptions: *Escherichia coli* K12 (Affymetrix, Santa Clara, CA); *Yersinia pestis*, *Franscisella tularensis*, *Burkholderia mallei*, *Brucella abortus*, *Bacillus anthracis* (BEI Resources, Manassas, VA); and Human, Arabidopsis and Rice (Zyagen, San Diego, CA).

### Preparation of genomic DNA Mix

Bacterial genomic DNA concentrations were determined using the Qubit dsDNA HS assay (Life Technologies). Bacterial genomes were diluted with water to obtain the desired concentrations and validated again using Qubit dsDNA HS assay before assembly of the final genomic DNA mix.

### HpaII gene cloning and transformation

*Haemophilus influenzae* was acquired from the American Type Culture Collection (ATCC® 49699™), and cultured in ATCC® Medium 814: GC Agar/Broth Medium (Teknova) at 37°C overnight with shaking. Total genomic DNA was isolated with the DNeasy Blood and Tissue Kit (Qiagen). The HpaII gene was amplified using forward primer GAGATATACCATGGCTGAATTTTTTTCTGGTAATAGAGG and reverse primer TCGAGGCTGCAGTTATAAGAATCTAATTTGTACGTTTAACTTAATAAAAAAATC (IDT, San Diego, CA) and the M. HpaII gene was amplified using forward primer AGATATACATATGAAAGATGTG TTAGATGATAA CTTGTTAG and reverse primer TCGAGGGTACCTCAGTCATATAAATTTCCTAATTTTTCT AAAATTTTCTTACCT (IDT, San Diego, CA). PCR was performed with Taq polymerase (Clontech) using the following cycle 95°C for 5 minutes, 40 cycles of (94°C for 15 seconds, 55°C for 15 seconds, 72°C for 1 minute), and 72°C for 5 minutes. The ~1100 bp HpaII PCR fragment was cloned using NcoI and PstI restriction sites in frame with the 5’ His tag of pETDuet-1 (EMD Millipore). The ~1100 bp M. HpaII PCR fragment was cloned using NdeI and KpnI into pACYCDuet-1 (EMD Millipore).

Recombinant vectors were isolated in 10-beta Competent *E*. *coli* cells (New England Biolabs). Co-transformations with pETDuet-1/HpaII and pACYCDuet-1/M. HpaII were executed in T7 Express Competent *E*. *coli* cells (New England Biolabs) by heat shock.

### HpaII protein purification and biotinylation

Expression and purification of His-HpaII protein was performed (MTIBIO, San Diego, CA) as follows: induction of the His-HpaII expressing *E*. *coli* was completed at an OD_600_ of 0.4–0.6 in a total volume of 20 L of LB at 37°C with 0.5 mM IPTG for 3 hours. Cell pellets were disrupted in lysis buffer (50 mM Tris-HCl, pH 7.5, 150 mM KCl, 20 mM imidazole, 0.5 mM TCEP, 5% glycerol) plus protease inhibitor cocktail (Sigma) using a microfluidizer. Following clarification by centrifugation (12,000g; 2 h; 4°C) the lysate was mixed with Ni-NTA superflow (Qiagen) for 2 h at 4°C, batch washed and transferred to a chromatography column that was subsequently equilibrated with lysis buffer. His-HpaII was eluted with a linear gradient of lysis buffer adjusted from 0 to 250 mM imidazole and 1 mL fractions collected. The fractions were analyzed by SDS-PAGE. Fractions 10 through 20 were pooled and dialyzed against 1L of 20 mM sodium phosphate pH 7.4, 500 mM NaCl at 4°C. The dialyzed protein was concentrated to approximately 4 mL (Amicon Ultra 15, 10000 MWCO; EMD Millipore) then further buffer exchanged on Sephadex G-25 columns (PD-10 columns; GE Healthcare) equilibrated with the same buffer to generate the final pool of approximately 3 mL. Activity of the purified recombinant His-HpaII was confirmed versus commercially available HpaII in a restriction digest of λ DNA (New England Biolabs).

His-HpaII was biotin labeled with the EZ-Link Sulfo-NHS-biotin kit (Pierce, Rockford, IL) following the manufacturer’s instruction. The extent of biotinylation was evaluated using the HABA assay (Pierce). Each mole of His-HpaII was found to contain 8.4 mole of biotin.

### HpaII mediated enrichment protocol

A 20 μl aliquot of streptavidin magnetic beads (New England Biolabs) was washed with once with 200 μl Buffer A (10 mM Tris pH 8.0, 50 mM NaCl, 10 mM CaCl_2_, 0.01% Tween 20) and resuspended in 50 μl of Buffer A containing 500 ng of biotinylated-His-HpaII. After pipette mixing to allow the His-HpaII to bind to the beads, the His-HpaII-beads (“HpaII-beads” for simplicity) were washed again with Buffer A. Enrichments were performed either in 1.7 ml microcentrifuge tubes or in a 96-well plate. DNA samples suspended in 50 μl of Buffer B (10 mM Tris pH 8.0, 250 mM NaCl, 10 mM CaCl_2_, 0.01% Tween 20) were added to HpaII-beads and mixed for the indicated time. Magnetic beads were separated using either a tube magnetic stand (Life Technologies) or a plate magnet (Millipore, Billerica, MA). The beads were washed once with 200 μl Buffer A, and then resuspended in 50 μl of Buffer B for qPCR analysis.

For gel analysis and next-generation library preparation, the DNA was eluted from beads by incubation with 50 μl 5 M guanidinium thiocyanate at room temperature for 5 minutes. The eluent was transferred to a 3,500 MWCO dialysis tube (Thermo Scientific, Waltham, MA) and dialyzed against distilled water for 1 hour at room temperature.

### McrB-N purification and biotinylation

A SalI-SacI fragment containing coding sequence for EcoKMcrB-N [18] was synthesized (GeneWiz) and cloned into the pET52 Expression Vector (Millipore) and transformed into the T7 Express cell line (NEB). The expressed recombinant protein has an N-teminal Strep tag and a C-terminal His tag from the pET52 vector to facilitate purification. Cultures were propagated at 37°C until OD_600_ is 0.4–0.6 and induced with IPTG at a final concentration of 0.05 mM. Induction was performed at 30°C on shaker for 4 hours and the cells were harvested by centrifugation. Lysates were prepared by Lysozyme treatment on ice and freeze-thaw. Lysates were clarified by centrifugation for 30 minutes followed by purification with Strep-Tactin Superflow Plus (Qiagen).

The tagged McrB-N was biotin labeled with the EZ-Link Sulfo-NHS-biotin kit (Pierce, Rockford, IL) following the manufacturer’s instructions. The extent of biotinylation was evaluated using the HABA assay (Pierce). Each mole of the tagged McrB-N was found to contain 6 mole of biotin.

### McrB-N enrichment protocol

700 ng tagged McrB-N-biotin was added to 50 ng of the Genomic DNA Mix in McrB-N Binding Buffer (10mM Tris pH7.5, 50 mM NaCl, 10mM CaCl2, 0.01% Tween20), mixed and incubated at 37°C for 1 hour. 80 μl of pre-washed streptavidin magnetic beads (NEB) were added, mixed and rotated at room temperature for 10 minutes. Magnetic beads were separated using a tube magnetic stand (Life Technologies). The supernatant (unbound fraction) was collected for analysis.

### Genomic DNA qPCR assay

Human RNaseP (Life Tech) and *Y*. *pestis* 3a sequence assay: forward -GGACGGCATCACGATTCTCT; reverse–CCTGAAAACTTGGCAGCAGTT (IDT); probe–AAACGCCCTCGAATCGCTGGC (Life Technologies) were used for quantification. Reactions were prepared using the QuantiProbe FAST PCR Kit (Qiagen) cycled once at 95°C for 3 minutes followed by 40 cycles of 95°C for 3 seconds and 60°C for 30 seconds on an ABI 7300. Relative abundance was calculated using either a standard curve or the delta Ct method.

### DNA isolation from sputum

A human sputum sample (BioreclamationIVT) was collected from at most 6 donors (pooled equally from 3 male and 3 female donors). Sputum was treated with an equal volume of 6.5 mM dithiothreitol (Sigma-Aldrich) for 30 minutes with occasional vortex mixing and was frozen in 0.5 ml aliquots. A 0.5 ml DTT-treated aliquot was thawed at room temperature. The DNeasy Blood and Tissue Kit was used to isolate DNA (Qiagen, Purification from Animal Tissues protocol). Briefly, 0.05 ml proteinase K and 0.5 ml Buffer AL was added to each sample and incubated at 56°C for at least 30 min with occasional mixing. A volume of 0.5 ml ethanol was added and the solution was loaded on the spin column up to three times. DNA was eluted twice with 30 μl 1X Binding Buffer [12] at 60°C and the eluates were combined. The DNA yield was determined with the Qubit BR assay (LifeTechnologies). A total of 5.3 μg of the extracted DNA was used for HpaII mediated enrichment protocol.

### Library preparation and sequencing

The Nextera DNA Sample Preparation Kit and Nextera XT DNA library Preparation Kit (Illumina, San Diego, CA) were used to prepare libraries from input, unbound, and bound/eluted fractions from HpaII and McrB-N mediated enrichment tests. Manufacturer’s instructions were followed for the library preparation except for recommended number of PCR cycles, which were varied according to the amount of DNA. For the genomic DNA mix, they were as follows: for 1:1,000 dilution samples: Input-9 cycles, HpaII bound-15 cycles, HpaII unbound-9 cycles. For 1:10,000 dilution samples: Input-9 cycles, HpaII bound-18 cycles, HpaII unbound-9 cycles. For 1:100,000 dilution samples: Input-9 cycles, HpaII bound-21 cycles, HpaII unbound-9 cycles. For the sputum sample: Input-9 cycles, HpaII bound-12 cycles, HpaII unbound-9 cycles. Libraries were sequenced following the manufacturer’s instructions for the HiSeq 2500 Rapid Run mode to obtain 50 nucleotide read lengths. The files corresponding to all the raw reads generated in this study are publicly available at the NCBI Short Read Archive (PRJNA287929).

### Metagenomic analysis

For microbial taxa identification, Illumina data sets were analyzed by an automated pipeline (ZovaSeq from Zova Systems, LLC, San Diego CA)[12] in which identifying sequence reads are assigned to specific microbial taxa when a given read length is found to occur uniquely within the taxa as defined by the NCBI taxonomy database [20, 21]. Relative abundance was calculated using two methods which gave equivalent results: tallying the number of ZovaSeq identifying reads or “microbial ID reads” for each bacterial taxa or by using Bowtie 1.0.0 to map reads to all identified organisms in the sample. For known higher eukaryotes in the sample (*Homo sapiens*, *Oryza sativa*) reads were mapped using Bowtie 1.0.0 with parameters allowing 2 mismatches in a 28 bp seed region.

Relative enrichment of the HpaII bound versus input samples were determined by the following “Bound/Input” equation:

$$Enrichment=\frac{BacterialREADCount_{BOUND}÷TotalREADCount_{BOUND}}{BacterialREADCount_{INPUT}÷TotalREADCount_{INPUT}}$$ Eq 1

Plots were generated by sequentially aligning sequence reads to all organisms included in the genome mixes, except for the organism for our organism of interest. The resulting unaligned reads were retained. The unaligned reads were then aligned to the organism of interest using default bowtie alignment options except for the following, the–e 4000 option was used to force only the consideration of the first 28 bp of each read. The resulting alignment file was opened in R (version 3.1.2) and coverage plots were generated by binning the total number bases covered in 5,000 bp increments and dividing by 5,000 to produce an average depth of coverage across each region.

## Results

### HpaII enriches *Y*. *pestis* genomic DNA from a Human DNA mixture

**Fig 1:**
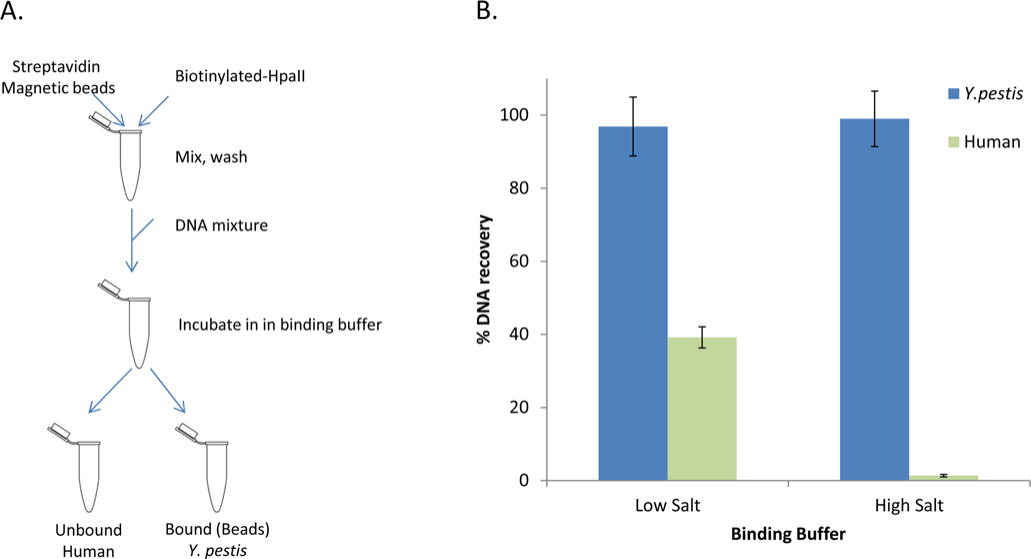
Enrichment workflow using HpaII. (A) Biotinylated HpaII enzyme is conjugated to streptavidin coated magnetic beads. A DNA mixture can then be added to the conjugated beads and following incubation the mixture is segregated into fractions that are bound (containing majority of *Y*. *pestis*) or unbound (containing majority of human) to the beads. (B) Adding salt to the binding buffer enhances segregation of human (blue bars) from *Y*. *pestis* (green bars).

HpaII protein was expressed and purified as described (S4 Fig). The obtained protein was biotinylated and endonuclease specificity was evaluated. HpaII-biotin cut *E*. *coli* genomic DNA into low molecular weight fragments (<500 bp) and showed little activity on human DNA (S1 Fig). To develop a magnetic bead based enrichment workflow, we removed magnesium ions from the reaction buffer which prevents digestion activity [12] but still enables HpaII to bind target DNA (Fig 1A). HpaII mediated enrichment conditions were optimized using selective qPCR assays on a predefined DNA mixture of *Yersenia pestis* and human genomes. We observed that increased salt during binding enhances differential binding of *Y*. *pestis* over human DNA (Fig 1B) with an optimal incubation time of 20 minutes.

**Fig 2:**
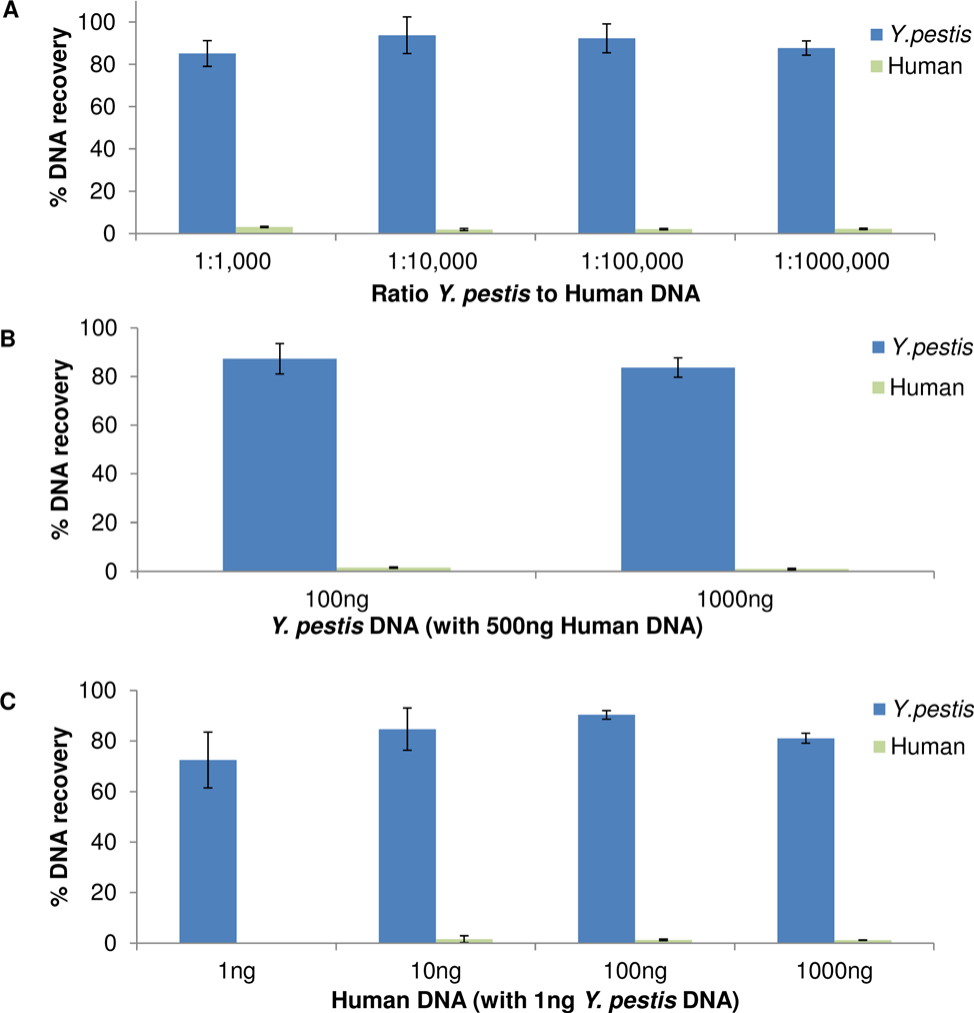
*Y*. *pestis* genomic DNA is efficiently segregated from human DNA. (A) Recovery of decreasing levels of *Y*. *pestis* DNA (blue bars) from a fixed 1000 ng human DNA (green bars). (B) DNA recovery using 100 ng and 1000 ng *Y*. *pestis* DNA in a background of 1000 ng human DNA. (C) Recovery of a fixed 1 ng of *Y*. *pestis* DNA (blue bars) from increasing levels of human DNA (green bars).

Genome mixtures composed of human DNA (fixed at 1 μg) with decreasing amounts of the *Y*. *pestis* genome (1 ng down to 1 pg) were used to test enrichment sensitivity. At a *Y*. *pestis* DNA to human DNA ratio of 1 pg:1 μg (1:10^6^), HpaII recovered over 80% of *Y*. *pestis* DNA while rejecting over 98% of human DNA (Fig 2A). Lower levels of *Y*. *pestis* DNA were not tested due to the limitation of the qPCR assay. We also observed that 20 μl of HpaII-beads can bind up to 1 μg *Y*. *pestis* DNA (Fig 2B). In our conditions HpaII capability to segregate *Y*. *pestis* DNA was examined in the presence of various levels of human DNA background. Less than 2% human DNA remained in enriched fractions (Fig 2C) when increasing human DNA (from 1 ng up to 1 μg) in the presence of 1 ng of *Y*. *pestis* genome which was retained at over 72%. These results demonstrate that HpaII can efficiently bind and segregate picogram quantities of *Y*. *pestis DNA* while rejecting microgram quantities of human DNA.

### HpaII enriches microbial DNA from human DNA background

To investigate the scope and efficiency of HpaII mediated enrichment, we mixed 1 pg of genomic DNA from each of a variety of organisms, including bacteria, plants (*Arabidopsis thaliana*, *Oryza sativa*), fungi (*Aspergillus* fumigatus and *Candida albicans*), and a parasite (*Cryptosporidium parvum*) in a background of human DNA. Thus, each genome is present at 1:100,000 ratio by mass relative to human DNA (Table 1).

**Table 1:** Genomic DNA mix contents in HpaII mediated enrichment test.

Domain	Type	Species	% by mass
Eukaryota	vertebrate	*Homo sapiens*	99.979
	plant	*Arabidopsis thaliana*	0.001
		*Oryza sativa*	0.001
	fungi	*Aspergillus fumigatus*	0.001
		*Candida albicans*	0.001
	parasite	*Cryptosporidium parvum*	0.001
Prokaryota	gram-negative	*Pseudomonas aeruginosa*	0.001
		*Yersinia pestis*	0.001
		*Burkholderia mallei*	0.001
		*Bordetella pertussis*	0.001
		*Bacterioides distasonis*	0.001
		*Neisseria gonorrhoeae*	0.001
		*Shigella flexneri*	0.001
		*Legionella pneumophila*	0.001
	gram-positive	*Bacillus anthracis*	0.001
		*Brucella abortus*	0.001
		*Staphylococcus aureus*	0.001
		*Mycobacterium tuberculosis*	0.001
		*Streptococcus pneumoniae*	0.001
	spirochete	*Borrelia burgdorferi*	0.001
Viruses	dsDNA virus	Human mastadenovirus C	0.001
		Orthopoxvirus vaccinia virus	0.001

**Fig 3:**
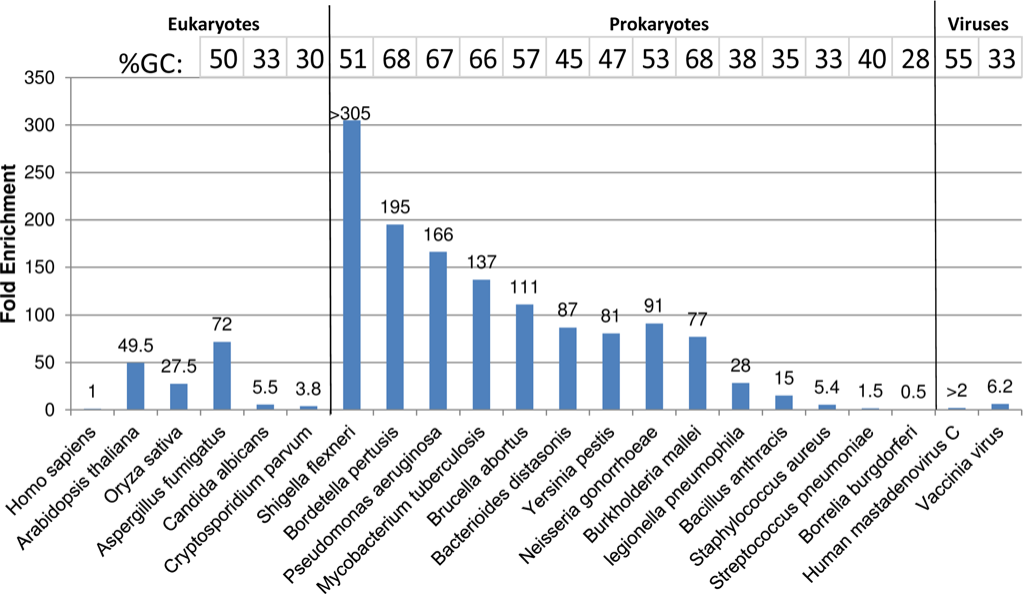
Sequence analysis of HpaII mediated enrichment of a genomic mixture. The fold enrichment (Eq 1) for each Eukaryote, Prokaryote and virus genome is listed. The GC content of microbial genomes are plotted above.

The mixture was subjected to the HpaII protocol and the input, unbound, and bound fractions were prepared for NGS. We observed different enrichment levels for individual microbial genomes (Fig 3). Most of the prokaryotic genomes enriched 70 to 200-fold. *S*. *flexneri*, *B*. *pertussis*, *P*. *aeruginosa*, *M*. *tuberculosis*, and *B*. *abortus* genomic DNA were all enriched over 100-fold (*Shigella* specific reads were undetectable in the input sample). *Bacterioides distasonis*, *Y*. *pestis*, *N*. *gonorrhoeae*, and *B*. *mallei* genomic DNA were enriched 70 to 100-fold. A few prokaryotic genomes were moderately enriched, such as those of *L*. *pneumophila* at 28-fold and *B*. *anthracis* at 15-fold. *S*. *aureus* and *S*. *pneumoniae* genomic DNA were slightly enriched at 5.4-fold and 1.5-fold respectively. *B*. *burgdorferi* was the only prokaryotic genome in this mixture where enrichment via HpaII was not observed. Among the DNA viruses tested, Vaccinia virus DNA enriched 6.2-fold, and Human Mastadenovirus C DNA was detectable only in the bound sample. *C*. *albicans* and *C*. *parvum* genomic DNA were slightly enriched at almost 5-fold, while plant genomes (*A*. *thaliana* and *O*. *sativa*) were both enriched over 20-fold. *A*. *fumigatus* genomic DNA was enriched 72-fold. Meanwhile, human mapped reads were lower in the bound fraction.

The differential enrichment of the tested microbial genomes was compared to the GC content of each as a surrogate for the frequency of unmethylated CCGG binding sites and their density. A relationship between the GC content of a genome and HpaII mediated enrichment levels was observed (Fig 3). HpaII mediated enrichment was repeated with the genomic DNA mixtures with microbial genomes present in increasing levels of human DNA at ratios of 1:100,000, 1:10,000 and 1:1,000 (S1 Table). A similar GC correlation pattern was observed. Microbial genome enrichment levels also showed improvement as the relative amount of human DNA increased.

**Fig 4:**
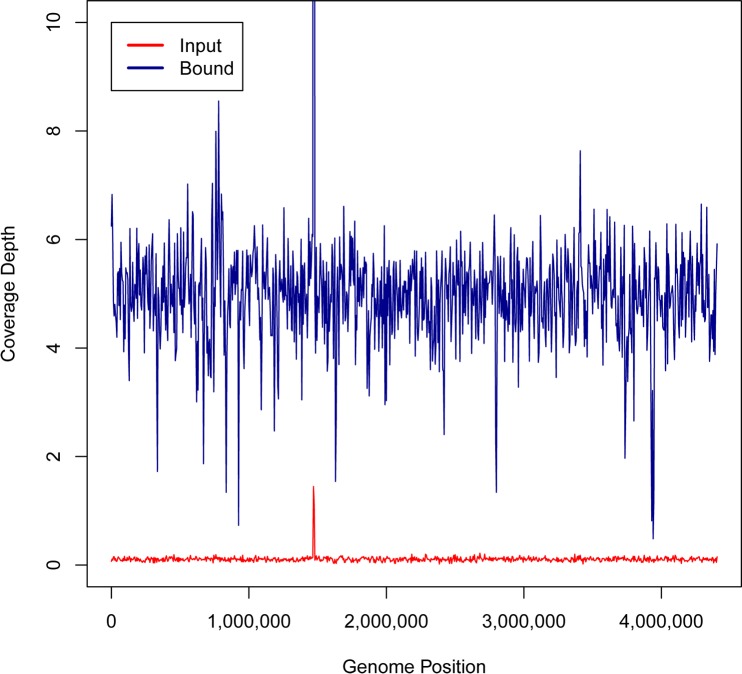
Genomic sequencing coverage of *M*. *tuberculosis* improves with enrichment. The input DNA sample coverage (red line) and HpaII bound coverage (blue line) are plotted across the genome position of *M*. *tuberculosis*.

HpaII mediated enrichment increased individual genome coverage. As an example, in the genomic DNA mix experiment, from the input fraction only 8.5% of the *M*. *tuberculosis* genome was sequenced at an average coverage depth of 0.09 (Fig 4). After HpaII mediated enrichment, 95.9% of the *M*. *tuberculosis* genome was sequenced, with an average coverage depth of 5.13 (Fig 4). Coverage improvements were also observed in the other microbial genomes in the mixture. An examination of *A*. *fumigatus* showed good coverage across all eight chromosomes (S2 Fig). *C*. *parvum* coverage was observed to be irregular (S3 Fig) for each of its eight chromosomes.

### HpaII enriches microbial genomic DNA from a sputum sample

**Fig 5:**
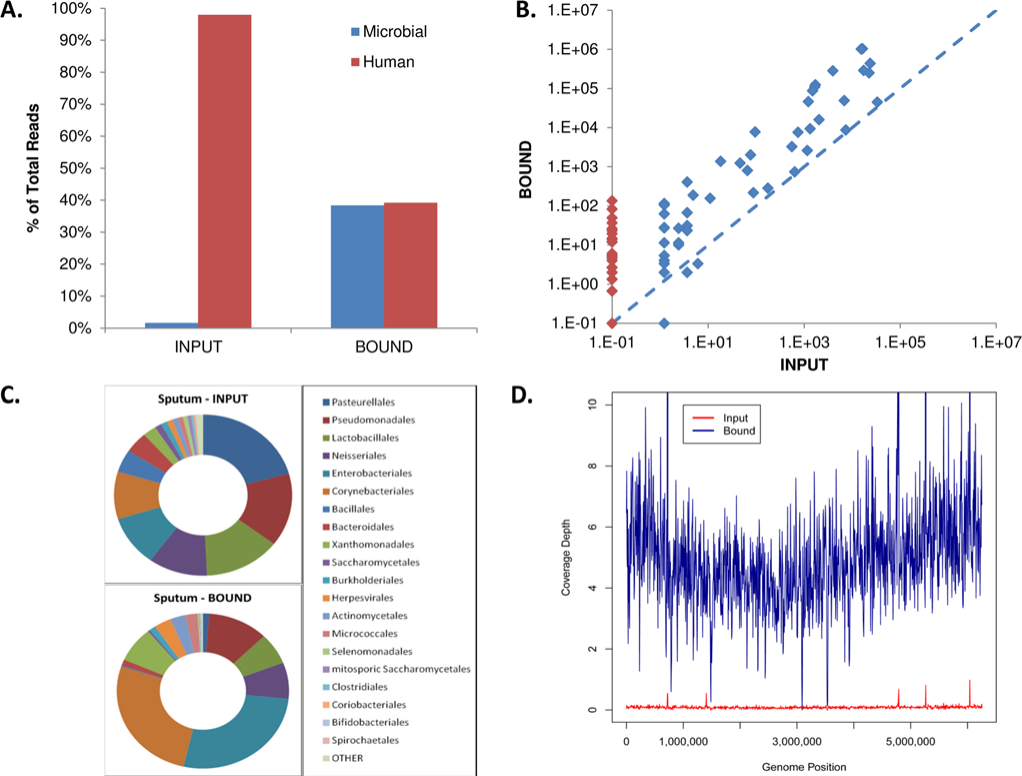
HpaII mediated enrichment of DNA from a pooled sputum sample improves microbe sequencing detection and coverage. (A) The percent of microbial ID reads (blue) increases and human ID reads (red) decrease with enrichment. (B) Normalized microbial Order sequence Identification reads are plotted for bound and input samples. Greater than 95% of identified microbes have increased sequenced reads. Many microbes (red points) are only detectable after enrichment. (C) Comparison of ratio of microbial sequence ID reads in sputum input and sputum bound samples. (D) Genomic sequencing coverage of bacteria such as *P*. *aeruginosa* improves with enrichment. The input DNA sample coverage (red line) and HpaII bound coverage (blue line) are plotted across the genome position of *P*. *aeruginosa*.

Our analysis of the pooled sputum sequencing data showed that 98% of the total sequencing reads mapped to human, with less than 2% microbial ID reads (Fig 5A). Following HpaII mediated enrichment, only 39.2% of the sequencing reads mapped to human while microbial ID reads increased to 38.4% of total reads (Fig 5A). Fig 5B shows that counts of microbial ID reads for nearly every Order increased in the bound fraction versus input; and several microbial Orders only had specific reads in the bound fraction (Fig 5B and Table 2).

**Table 2:** HpaII mediated enrichment levels of microbial genera from sputum.

Genus	B/I*	Bound Reads**	Genus	B/I*	Bound Reads	Genus	B/I*	Bound Reads
*Lymphocryptovirus*	401	489	*Yersinia*	60	800	*Bulleidia*	21	510
*Morganella*	239	291	*Rothia*	58	84,047	*Cronobacter*	19	93
*Arcanobacterium*	>195	195	*Dialister*	58	922	*Pyramidobacter*	19	92
*Sodalis*	141	344	*Actinomyces*	58	26,689	*Edwardsiella*	18	86
*Cupriavidus*	130	158	*Pseudoramibacter*	56	137	*Eubacterium*	17	383
*Parascardovia*	119	580	*Slackia*	56	272	*Neisseria*	17	251,660
*Shuttleworthia*	111	407	*Lautropia*	55	335	*Aggregatibacter*	16	1,325
*Mastadenovirus*	108	396	*Enterobacter*	55	39,473	*Phikzlikevirus*	16	326
*Ralstonia*	103	125	*Myxococcus*	>54	54	*Abiotrophia*	15	110
*Jonquetella*	>96	96	*Salmonella*	53	321	*Filifactor*	12	511
*Bacteroides*	95	7,174	*Oribacterium*	52	2,809	*Proteus*	10	11,941
*Dechloromonas*	>93	93	*Tropheryma*	51	877	*Nakaseomyces*	7.9	14,775
*Acidovorax*	>90	90	*Moniliophthora*	>50	50	*Acinetobacter*	7.2	8,181
*Propionibacterium*	87	1,374	*Escherichia*	45	38,144	*Solobacterium*	6.1	240
*Cardiobacterium*	86	105	*Variovorax*	43	52	*Anaerococcus*	5.6	55
*Dickeya*	>85	85	*Comamonas*	42	51	*Atopobium*	4.5	2,382
*Serratia*	82	17,681	*Lambdalikevirus*	41	50	*Veillonella*	4.5	5,363
*Klebsiella*	80	436,667	*Catonella*	39	1,523	*Parvimonas*	4.2	679
*Bifidobacterium*	80	6,914	*Eikenella*	38	3,651	*Providencia*	4.1	974
*Penicillium*	>78	78	*Lactobacillus*	34	209,551	*Prevotella*	3.9	23,267
*Simplexvirus*	72	116,770	*Janthinobacterium*	33	3,523	*Granulicatella*	2.8	719
*Stenotrophomonas*	71	226,921	*Porphyromonas*	33	15,455	*Peptostreptococcus*	2.6	183
*Herbaspirillum*	70	426	*Pseudomonas*	33	402,158	*Campylobacter*	2.4	215
*Bacillus*	70	254	*Olsenella*	33	160	*Streptococcus*	2.4	32,196
*Mycobacterium*	69	418	*Citrobacter*	33	1,746	*Candida*	2.2	2,600
*Bordetella*	68	2,499	*Mobiluncus*	31	76	*Capnocytophaga*	1.5	252
*Pantoea*	>68	68	*Cryptobacterium*	31	265	*Enterococcus*	1.4	2,349
*Scardovia*	>68	68	*Megasphaera*	31	1,487	*Haemophilus*	1.2	33,624
*Corynebacterium*	66	1,015,615	*Burkholderia*	30	13,924	*Fusobacterium*	1.2	719
*Erwinia*	65	79	*Kingella*	29	991	*Staphylococcus*	1.2	7,044
*Achromobacter*	63	3,942	*Treponema*	26	1,982	*Gemella*	0.8	993
*Herminiimonas*	63	154	*Alloprevotella*	26	537	*Peptoniphilus*	0.5	91
*Pectobacterium*	>62	62	*Delftia*	24	3,797	*Moraxella*	0.5	4,351
*Xanthomonas*	60	364	*Selenomonas*	24	1,380	* *		

“*” B/I (see Eq 1)

“**”Bound Reads is number of normalized HpaII bound sequence reads

“>” Indicates there were no input reads.

Pasteurellales, Actinomycetales, Enterobacteriales, Pseudomonadales, Lactobacillales, and Neisseriales constitute the majority of the microbial orders identified in the sputum sample (Fig 5C). After HpaII mediated enrichment, Actinomycetales and Enterobacteriales are the two major orders identified in the bound fraction. The normalized total microbial ID reads increased from 161,942 reads in the input fraction to 3,837,809 reads in the bound fraction. The enrichment levels of different microbes are listed in Table 2. The identified microbial genera can be grouped into 4 categories: highly enriched (>50-fold), moderately enriched (10 to 50-fold), slightly enriched (<10-fold), and reduced (≤1-fold). The majority of the identifiable microbial genera fall into either the highly enriched category or the moderately enriched category (58 out of 82) (Table 2), among them are clinically relevant pathogens such as *Mycobacteria* and Herpesvirus. Consistent with previous observations (Fig 3B) the majority of the enriched genera have an average GC content over 40%, while the non-enriched or slightly enriched groups generally contain less than 40% GC in their genomes.

Microbial genome coverage also improved with HpaII mediated enrichment from sputum (Fig 5D). For example prior to enrichment 5.2% of the *P*. *aeruginosa* genome was sequenced at an average coverage depth of 0.06. Following HpaII binding, 93.1% of the genome was sequenced to an average depth of 4.6.

### McrB-N enriches microbial genomes via specific binding to human genome

**Fig 6:**
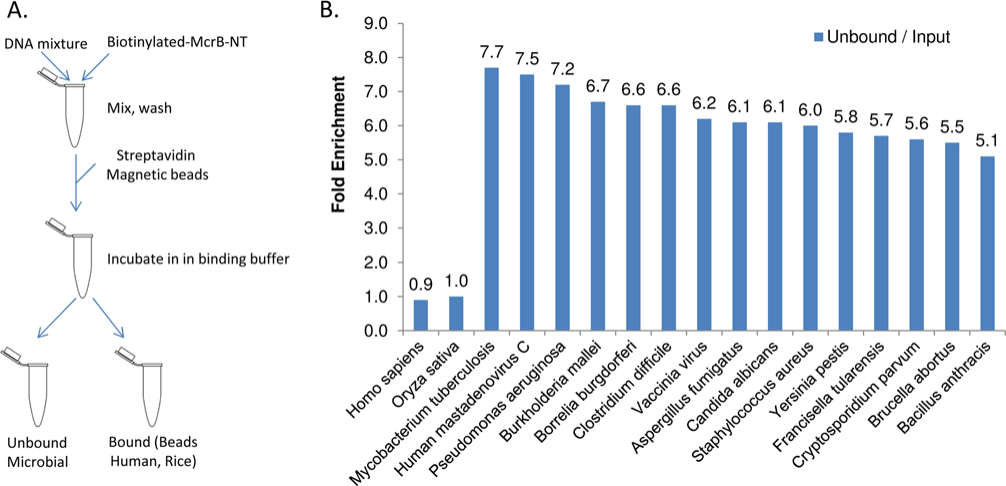
Enrichment workflow using McrB-N. (A) Biotinylated McrB-N enzyme is added to a DNA mixture. Following the addition of strepatavidin coated magnetic beads, the mixture is segregated into fractions that are bound (containing majority of human) or unbound (containing majority of microbes). (B) Sequence analysis demonstrates that McrB-N segregates human and rice DNA away from microbial genomes in the unbound fraction. The fold enrichment for each taxa is plotted for the unbound (blue) fraction.

We expressed and purified the N-terminal DNA-binding domain of McrB from the Type IV endonuclease McrBC (S5 Fig). The purified fragment, which lacks restriction activity, was biotinylated and tested for its ability to differentially bind methyl CpG motifs commonly found in human DNA. When added to a genomic mixture containing bacteria, dsDNA viruses, and fungi at 1/1000 dilution with human and rice genomes (S2 Table) we observed that all microbial genomic DNA was enriched approximately 5 to 8-fold in the unbound fraction, relative to human and rice (Fig 6). The relative ratios of the enriched non-bound genomic DNA tested remains intact demonstrating the utility of a Type IV enzyme for selective enrichment.

## Discussion

To segregate bacterial genomic DNA from host backgrounds, selective enrichment protocols were developed using the Type II restriction endonuclease HpaII and a fragment of the Type IV restriction endonuclease McrB. HpaII recognizes unmethylated CCGG sequences and is blocked by the methylated C^m^CGG motif. Since CpG methylation occurs frequently in eukaryotic genomes (the majority of CCGG sites are methylated in human [22]), we hypothesized that HpaII would specifically bind and concentrate microbial genomic DNA, which have lower levels of m5C [16, 23], from mixtures containing human and higher eukaryotic genomic DNA. Conversely, McrB binds DNA sequences containing methylated CpG [18]; thus we used the McrB binding domain as the basis to develop a tool that selectively binds human DNA. Using these two strategies, we examined enrichment profiles in genomic DNA mixtures.

HpaII demonstrated efficient segregation of the *Y*. *pestis* genome from human DNA at a 1,000,000-fold mass excess (Fig 2A and 2B). Removal of human DNA (> 95%) and target DNA retention (>80% *Y*. *pestis* DNA recovery) gave high enrichment levels. In genomic DNA mixtures, HpaII mediated enrichment improved the read coverage of all bacterial DNA tested except *Borrelia burgdorferi* (Fig 3A, S1 Table). It has been observed that *B*. *burgdorferi* transformation efficiency improves after *in situ* CpG methylation of plasmid DNA [24]. This implies that the *B*. *burgdorferi* genome contains methylated CpG motifs which would be consistent with the reduced HpaII mediated enrichment we observed. In sputum samples, virtually all bacterial genomes identified were enriched (Fig 5), some greater than 100-fold. Many genomes were observable only after HpaII mediated enrichment.

Differences in the level of enrichment seem to be loosely related to the GC content of the bacterial genome (Fig 3B). We anticipate this is related to the number and density of CCGG sites and the absence of overlapping cytosine methylation. A consequence of this “GC” bias is that HpaII mediated enrichment does not preserve the ratio of microbial DNA in a mixture as McrB-N does, but the over 50-fold enrichment of organisms such as *Mycobacteria*, and *Bordetella* dramatically improves detection and organism coverage by NGS methods (Figs 4 and 5D). Of course the GC content relationship to enrichment is not absolute due to methylome differences as is the case for *B*. *burgdorferi*.

Epigenetic enrichment suggested interesting features of the genomes we tested. For instance, fungi display a large range of m5C content in their genomes [25] and we saw differing enrichment results for *Candida albicans* and *Aspergillus fumigatus* (Fig 3A, S1 Table). Generally, fungal genomes are hypomethylated compared to higher eukaryotic genomes [25]. Studies based on bisulfite sequencing and methyltransferase analyses demonstrate that DNA methylation is largely absent in *Aspergillus* families [26, 27] which would explain the 72-fold enrichment of *A*. *fumigatus* we observed with HpaII (Fig 3A). Coverage of the *A*. *fumigatus* genome was improved and fairly even across all 8 chromosomes supporting the idea that little of its genome is methylated at CCGG sites (S2 Fig). In contrast, the dimorphic yeast *C*. *albicans* uses cytosine methylation to modulate the transition between yeast and hyphal forms among other transcription events [28]. The presence of CpG methylation in *C*. *albicans* correlates with the lower genome enrichment of 5.5 fold relative to that of *A*. *fumigatus* (Fig 3A).

Another eukaryotic genome in our genomic mix, *C*. *parvum*, has poorly characterized epigenetic patterns. *C*. *parvum* has a complex, monoxenous life cycle consisting of several developmental stages involving both sexual and asexual cycles [29] and poorly understood gene regulation mechanisms [30] all of which are candidates for epigenetic regulation. The *C*. *parvum* genome encodes one protein with similarity to the Dnmt2 family, which is responsible for DNA methylation at cytosines in *Entamoeba*, mainly at repetitive elements and retrotranposons [30]. Isolation of purified DNA from *C*. *parvum* suitable for NGS is a time consuming and challenging process particularly from natural samples such as stool. Current best practices involve rounds of oocyte purification and whole genome amplification which still leave contamination from host, bacterial and digestive content genomes [31]. *C*. *parvum* has been evaluated for methylated cytosine using mass spectroscopy and none was detected to a sensitivity of less than 0.04% [30]. Thus any sequence targets for the putative *C*. *parvum* cytosine methylatse remain unknown if any exist. Not surprisingly, *C*. *parvum* DNA was enriched in the microbial fraction by McrB-N consistent with the absence or low levels of CpG methylation (Fig 6). Thus McrB-N offers utility as a tool to improve isolation and enrichment of Cryptosporidium DNA for whole-genome sequencing. HpaII mediated enrichment did show a slight preference for *C*. *parvum* (3.8-fold) relative to human genomic DNA (Fig 3) but not the high enrichment seen with the other non-methylated cytosine organisms. This suggests that there are differences in the *C*. *parvum* methylome compared to the other microbial organisms we have tested. Genomic coverage *of C*. *parvum* was uneven (S3 Fig). An analysis of genomic content enriched by HpaII is ongoing.

Human sputum is commonly used as a noninvasive diagnostic tool, however sequencing analysis of microbial contents of sputum is challenging mainly due to the presence of high levels of human DNA. Indeed, 98% of our sputum sequencing data prior to HpaII mediated enrichment was attributed to human DNA (Fig 5A); after enrichment by HpaII half the annotated reads were microbial (Fig 5A). Moreover, nearly all of the identified microbial genera DNA was enriched by HpaII (98 of 101 genera, Table 2). This includes known pathogens such as *Mycobacterium tuberculosis* (69-fold enriched). Although the current study and samples were not set up for assessing drug resistance, the sequencing improvement in most of the microbes would allow SNP/SNV calling that would be informative for pathogens like *M*. *tuberculosis*. We are encouraged that HpaII functions most efficiently in the presence of high levels of clutter DNA (S1 Table). Therefore, clinical samples with high human background such as blood and saliva may also be suitable for HpaII treatment prior to NGS analysis to enhance diagnostic sensitivity. In concept, the increased sequencing reads and improved genome coverage from HpaII mediated enrichment would enable the detection of trace or unculturable microbes, identification of novel species/strains, and characterization of virulent and resistant attributes of pathogens.

Double stranded DNA viral genomes were enriched by HpaII in both the genomic DNA mixture and sputum samples (Fig 3A, S1 Table and Table 2) and remained in the microbial DNA unbound fraction with McrB-N (Fig 6). Cytosine methylation in DNA viruses demonstrates complexity in relation to the genome replication state and host environment [32]. For instance, alpha-herpesvirinae and gamma-herpesvirinae are hypomethylated during active replication although their methylation status during latency is unknown [32]. Others have reported detecting oncoviruses including EBV and HPV in CpG enriched sequencing data of cervical samples, supporting the idea that these viruses are methylated in these samples [33]. In our genomic DNA mixture, Vaccinia virus and human mastadenovirus C genomes were slightly enriched (Fig 3A). In sputum, lymphocryptovirus, mastadenovirus and simplexvirus genomes were all enriched over 70-fold, and Cytomegalovirus over 20-fold (Table 2). The results suggest that these viral genomes are all highly methylated. This is consistent with current research and supports epigenetic enrichment as a functional tool for the detection of some DNA viruses, with potential utility for the analysis of viral replication states.

Plant genomes possess complex patterns of methylation [34–36]. Unlike animal genomes where m5C is predominantly found in CG motifs, cytosines in plant DNA have been reported as methylated in mCCGG, CmCGG, and mCmCGG motifs [37–39]. HpaII has no restriction activity on CmCGG and little or no activity on hemi-methylated CCGG variants [40, 41]. The two plant genomes in our genomic DNA mixture were moderately enriched by HpaII (Fig 3A) relative to human. We postulate this is likely because plant CCGG sequences are not methylated in the inner cytosine. The rice genome was reported to contain a higher frequency of DNA methylation than Arabidopsis [42], consistent with their relative enrichment levels in our HpaII results (Fig 3A). Epigenetic removal of rice DNA was also efficient with McrB-N (Fig 6).

Each epigenetic strategy presented has advantages. For instance, since McrB-N binds and removes CpG containing i.e. typical host genomes, no elution is needed to recover the microbial fraction. This minimizes time and sample loss although the output volume will be approximately equivalent to the input. HpaII, on the other hand binds microbial genomes without a CmCGG motif. This allows elution of the microbial fraction in a determined volume providing a concentration step. Furthermore while the microbial fraction is bound to magnetic beads, we find that extensive washing can remove impurities that would otherwise be present in the sample.

Type IV restriction enzymes are a group of modification-dependent restriction endonucleases with representative enzymes that discriminate methylated motifs such as 5-methylcytosine, hydroxyl-5-methylcytosine and glucosylhydroxy-5-methylcytosine among other DNA modifications [43, 44]. McrB-N is the first used to segregate the CpG methylomes of human and plant from microbial genomes (Fig 6). Unlike Type II endonucleases, binding and restriction functions are separated into different protein subunits. McrB forms heptameric rings as well as tetradecamers with a central channel in the presence of Mg^++^ and GTP [19]. In the presence of McrC, the DNA cleavage subunit, the tetradecameric species is the major form of the endonuclease. We did not test the extent to which intact McrB or addition of McrC improves enrichment. Our results suggest the use of other Type IV restriction endonucleases may be useful in enriching other DNA methylation patterns of interest.

This work demonstrates the development of two restriction endonucleases for epigenetic enrichment with respect to the presence of CpG motifs. The specificity of restriction endonucleases in discriminating methylated DNA makes them efficient tools to segregate genomic mixtures into target methylomes. The majority of bacterial, viral, fungal and protist genomes that we tested were enriched by this approach, improving detection, coverage and insights into the genomic methylation state of the organism. Our test of sputum revealed enhanced enrichment of genomic DNA from target pathogens such as *M*. *tuberculosis* and some DNA viruses from a background of human DNA. Testing is still underway to determine if subsets of viral or protist DNA collected at different life cycle stages are preferentially collected. However strategies to differentiate epigenetic states that can occur during replication, differentiation, transcription, cancer and host pathogen interactions are easily envisioned. The expanding set of epigenetic tools and in particular restriction enzymes that discriminate N-6-methyl adenine [12] and C-5-methyl cytosine [45] should facilitate the analysis of methylated genomes and epigenetic patterns across the biological kingdoms.

## Supporting Information

S1 Fig Biotinylated HpaII shows specific restriction activity. Biotinylated HpaII was used to digest (+) various genomic templates, or run without HpaII digestion (-). CCGG Unmethylated genomes (plasmid pXYLT5, *E*. *coli* and Bacilli) are cut by HpaII, while methylated pXylT5 (mCG) and human remain uncut.
(TIF)

S2 Fig *A*. *fumigatus* Af293 sequence coverage in bound (blue) and input (red) samples. Each chromosome is labeled as 1–8. Genome position in base pairs is shown on the horizontal axis and coverage depth is plotted on the vertical axis as shown for chromosome 1. Noticeable gaps on each chromosome correspond with centromere locations and the ~250 KB gap starting at approximately 450,000 bp on chromosome 4 corresponds with the gap in the NCBI genomic sequence for the ribosomal DNA repeat region. Chromosome 4 is thus scaled equivalent to other plots to facilitate viewing.
(TIF)

S3 Fig Cryptosporidium parvum Iowa IILP326 sequence coverage in bound (blue) and input (red) samples. Each chromosome is labeled as 1–8. Genome position in base pairs is shown on the horizontal axis and coverage depth is plotted on the vertical axis as shown for chromosome 1.
(TIF)

S4 Fig SDS-PAGE analysis of even numbered Ni-NTA HpaII fractions. Comparison to the molecular weight marker (MW) shows the expected band of 36 KD (red arrow). Fractions 8 through 28 were pooled (red line) to generate the material for use.
(TIF)

S5 Fig McrB-NT protein purification. Culture before (T0) and 4 hours post induction (T4), lysate, pellet, flow through, wash, and Strep-Tactin Superflow Plus elutions (2–8) were run on a 14% acrylamide Tris-Glycine gel. A protein of a size consistent with McrB-NT (red arrow) is observed in the post induction culture and in the cell lysate. Elutions 4–6 were pooled to generate the material for use.
(TIF)

S1 Table HpaII mediated enrichment at various genome dilutions. (DOCX)

S2 Table Genomic mixture contents in HpaII mediated enrichment test. (DOCX)
