# Supplementary figures and images for: Epigenetic Segregation of Microbial Genomes from Complex Samples Using Restriction Endonucleases HpaII and McrB
Source: PLoS One. 2016 Jan 4;11(1):e0146064. doi: 10.1371/journal.pone.0146064 (PMC4699840; doi:10.1371/journal.pone.0146064)

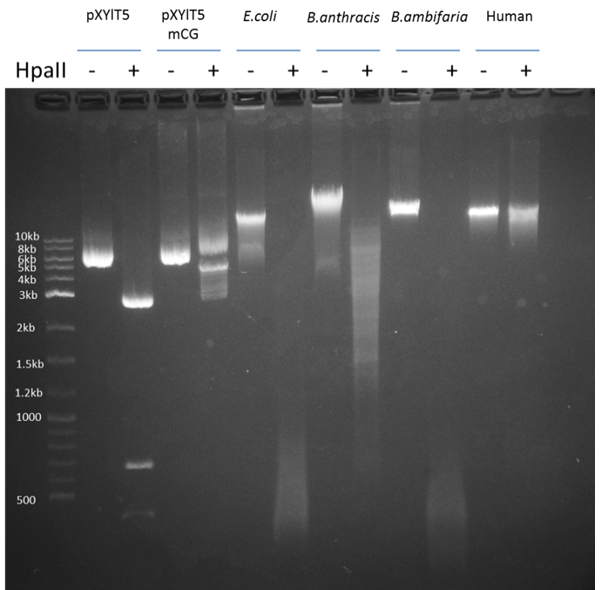

Supplement: S1 Fig — Biotinylated HpaII was used to digest (+) various genomic templates, or run without HpaII digestion (-). CCGG Unmethylated genomes (plasmid pXYLT5, E. coli and Bacilli) are cut by HpaII, while methylated pXylT5 (mCG) and human remain uncut. (TIF) [file pone.0146064.s001.tif]

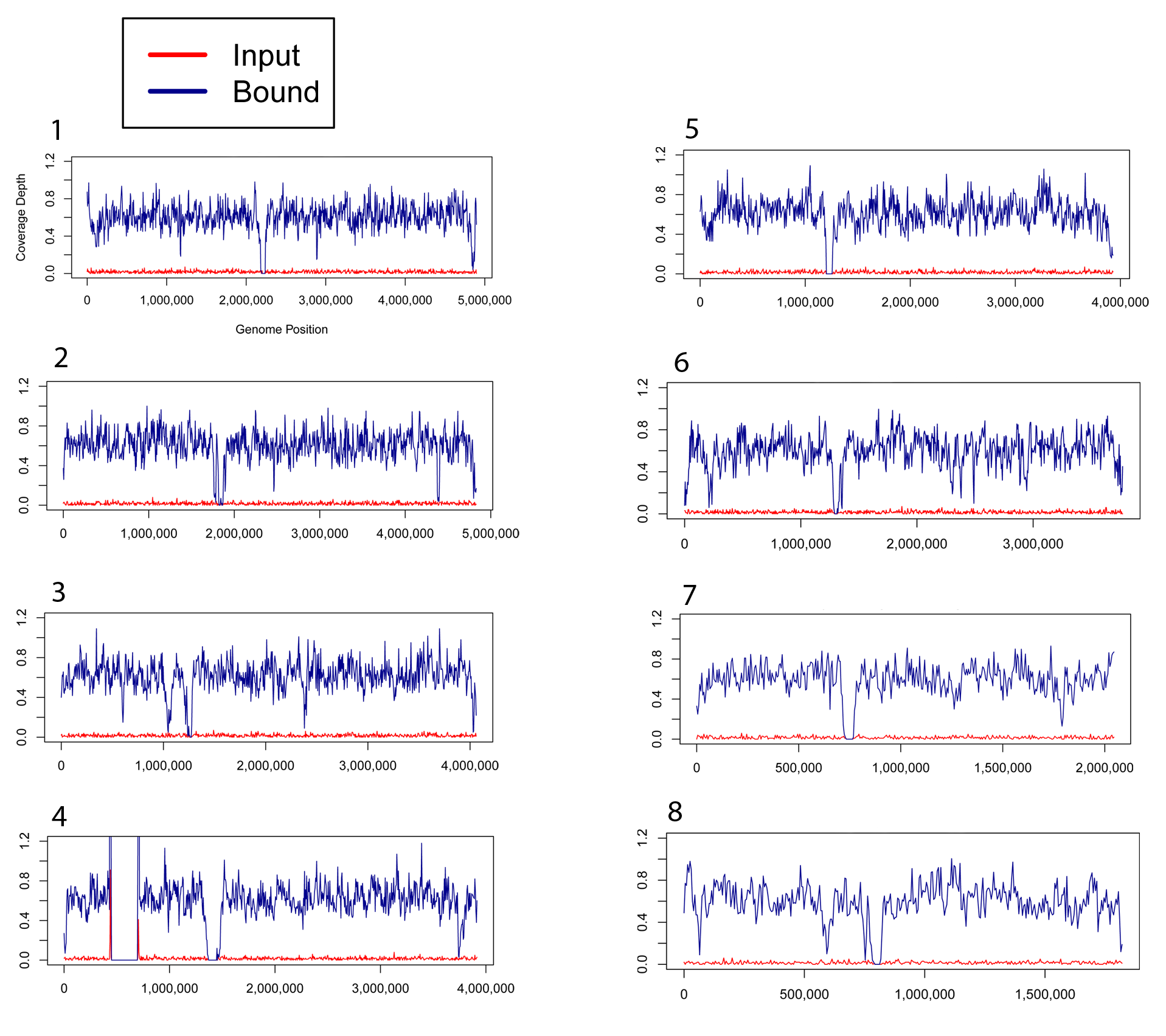

Supplement: S2 Fig — Each chromosome is labeled as 1–8. Genome position in base pairs is shown on the horizontal axis and coverage depth is plotted on the vertical axis as shown for chromosome 1. Noticeable gaps on each chromosome correspond with centromere locations and the ~250 KB gap starting at approximately 450,000 bp on chromosome 4 corresponds with the gap in the NCBI genomic sequence for the ribosomal DNA repeat region. Chromosome 4 is thus scaled equivalent to other plots to facilitate viewing. (TIF) [file pone.0146064.s002.tif]

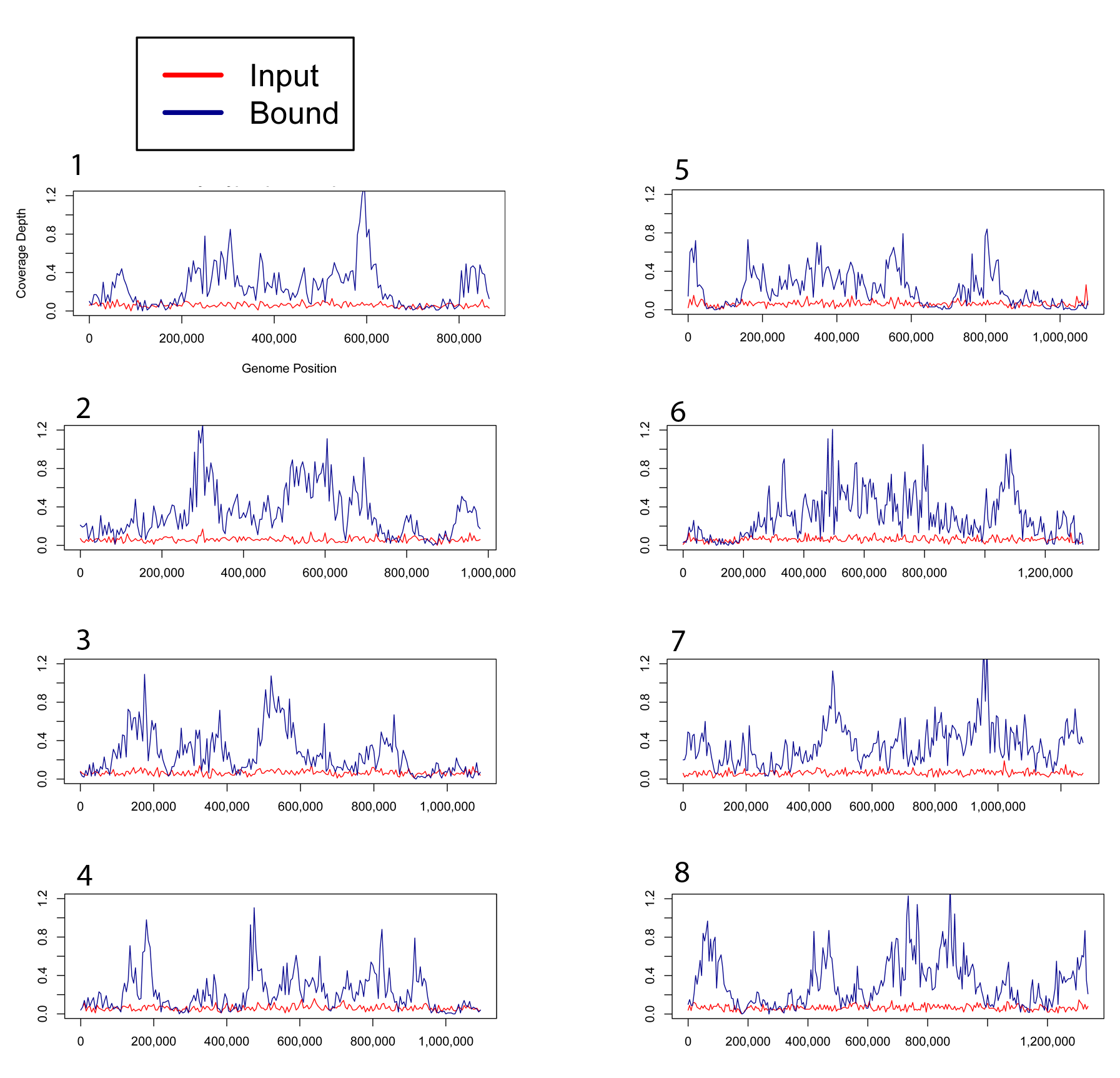

Supplement: S3 Fig — Each chromosome is labeled as 1–8. Genome position in base pairs is shown on the horizontal axis and coverage depth is plotted on the vertical axis as shown for chromosome 1. (TIF) [file pone.0146064.s003.tif]

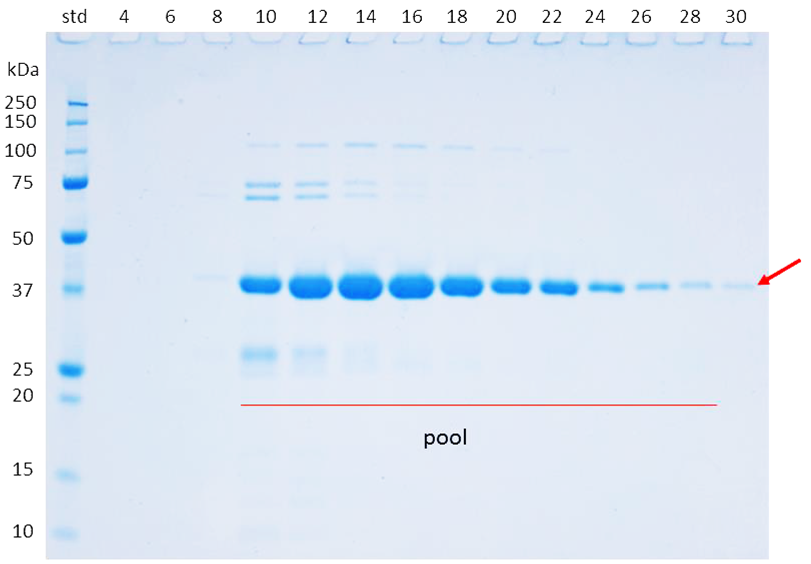

Supplement: S4 Fig — Comparison to the molecular weight marker (MW) shows the expected band of 36 KD (red arrow). Fractions 8 through 28 were pooled (red line) to generate the material for use. (TIF) [file pone.0146064.s004.tif]

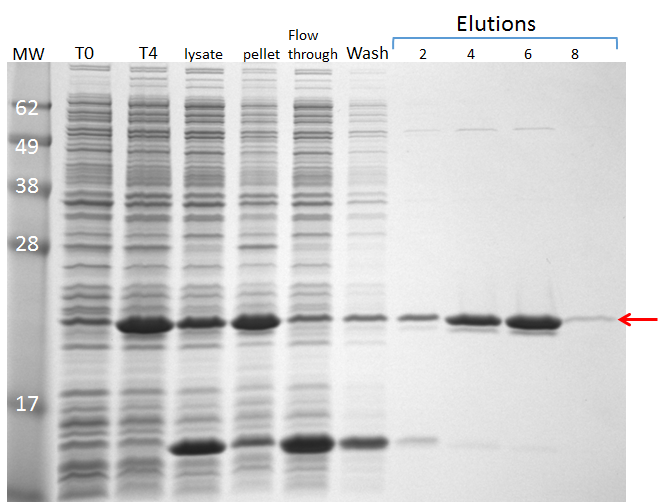

Supplement: S5 Fig — Culture before (T0) and 4 hours post induction (T4), lysate, pellet, flow through, wash, and Strep-Tactin Superflow Plus elutions (2–8) were run on a 14% acrylamide Tris-Glycine gel. A protein of a size consistent with McrB-NT (red arrow) is observed in the post induction culture and in the cell lysate. Elutions 4–6 were pooled to generate the material for use. (TIF) [file pone.0146064.s005.tif]
