# Supplementary material for: Epigenetic Segregation of Microbial Genomes from Complex Samples Using Restriction Endonucleases HpaII and McrB
Source: PLoS One. 2016 Jan 4;11(1):e0146064. doi: 10.1371/journal.pone.0146064 (PMC4699840; doi:10.1371/journal.pone.0146064)
Supplement: S1 Table — (DOCX) [file pone.0146064.s006.docx]

**S1 Table**. HpaII mediated enrichment at various genome dilutions

|  |  | **Bound / Input** | | |
| --- | --- | --- | --- | --- |
|  |  | **1/1000** | **1/10,000** | **1/100,000** |
| Eukaryota | *Homo sapiens* | 0.3 | 0.8 | 1.0 |
|  | *Arabidopsis thaliana* | 7.0 | 27.7 | 49.5 |
|  | *Oryza sativa* | 6.0 | 21.0 | 27.5 |
|  | *Aspergillus fumigatus* | 33.7 | 66.5 | 71.7 |
|  | *Candida albicans* | 3.9 | 7.4 | 5.5 |
|  | *Cryptosporidium parvum* | 4.7 | 7.6 | 3.8 |
| Prokaryota | *Shigella flexneri* | 53.5 | 72.1 | >305 |
|  | *Bordetella pertussis* | 36.3 | 93.5 | 195.1 |
|  | *Pseudomonas aeruginosa* | 43.4 | 96.0 | 166.0 |
|  | *Mycobacterium tuberculosis* | 44.8 | 112.7 | 136.8 |
|  | *Brucella abortus* | 52.5 | 89.9 | 110.6 |
|  | *Bacterioides distasonis* | 56.0 | 81.8 | 86.8 |
|  | *Yersinia pestis* | 50.7 | 81.6 | 80.7 |
|  | *Neisseria gonorrhoeae* | 43.8 | 69.7 | 91.1 |
|  | *Burkholderia mallei* | 29.1 | 77.1 | 76.9 |
|  | *Legionella pneumophila* | 26.1 | 58.5 | 28.3 |
|  | *Bacillus anthracis* | 15.2 | 23.2 | 15.0 |
|  | *Staphylococcus aureus* | 2.7 | 6.4 | 5.4 |
|  | *Streptococcus pneumoniae* | 0.3 | 1.6 | 1.5 |
|  | *Borrelia burgdorferi* | 0.5 | 1.0 | 0.5 |
| Viruses | Human mastadenovirus C | 52.2 | >25 | >2.0 |
|  | Vaccinia virus | 8.2 | 15.4 | 6.2 |

“>” Indicates there were no input reads.
