# Supplementary material for: Epigenetic Segregation of Microbial Genomes from Complex Samples Using Restriction Endonucleases HpaII and McrB
Source: PLoS One. 2016 Jan 4;11(1):e0146064. doi: 10.1371/journal.pone.0146064 (PMC4699840; doi:10.1371/journal.pone.0146064)
Supplement: S2 Table — (DOCX) [file pone.0146064.s007.docx]

**S2 Table**. Genomic mixture contents in HpaII mediated enrichment test

| **Domain** | **Type** | **Species** | **% by mass** |
| --- | --- | --- | --- |
| Eukaryota | clutter | *Homo sapiens* | 98.4 |
|  | clutter/plant | *Oryza sativa* | 0.1 |
|  | fungal | *Aspergillus fumigatus* | 0.1 |
|  | fungal | *Candida albicans* | 0.1 |
|  | parasite | *Cryptosporidium parvum* | 0.1 |
| Prokaryota | gram-negative | *Burkholderia mallei* | 0.1 |
|  | gram-negative | *Pseudomonas aeruginosa* | 0.1 |
|  | gram-negative | *Yersinia pestis* | 0.1 |
|  | gram-positive | *Bacillus anthracis* | 0.1 |
|  | gram-positive | *Brucella abortus* | 0.1 |
|  | gram-positive | *Clostridium difficile* | 0.1 |
|  | gram-positive | *Francisella tularensis* | 0.1 |
|  | gram-positive | *Mycobacterium tuberculosis* | 0.1 |
|  | gram-positive | *Staphylococcus aureus* | 0.1 |
|  | spirochete | *Borrelia burgdorferi* | 0.1 |
| Viruses | dsDNA virus | *Human mastadenovirus C* | 0.1 |
|  | dsDNA virus | *Vaccinia virus* | 0.1 |
